# Observational longitudinal study on *Toxoplasma gondii* infection in fattening beef cattle: serology and associated haematological findings

**DOI:** 10.1007/s00436-024-08189-y

**Published:** 2024-03-23

**Authors:** Filippo M. Dini, Joana G. P. Jacinto, Damiano Cavallini, Andrea Beltrame, Flavia S. Del Re, Laura Abram, Arcangelo Gentile, Roberta Galuppi

**Affiliations:** 1 Department of Veterinary Medical Sciences, Alma Mater Studiorum University of Bologna, Via Tolara Di Sopra 50, 40064 Ozzano Emilia, BO Italy; 2 Clinic for Ruminants, Vetsuisse Faculty, University of Bern, 3012 Bern, Switzerland; 3 Institute of Genetics, Vetsuisse Faculty, University of Bern, 3012 Bern, Switzerland; 4 Verona, Italy

**Keywords:** Antibody, Bovine, *T. gondii*, IFAT, Haematology, Neutrophil

## Abstract

Toxoplasmosis, caused by the protozoan parasite *Toxoplasma gondii*, is a globally distributed zoonotic infection with significant implications for human and animal health. This study investigated the prevalence of *T. gondii* infection in a population of beef cattle at three different stages of their productive lifespan and examined the impact of *T. gondii* serological status on blood parameters. A commercial beef fattening unit in Italy was the setting for this research, which involved a biosecurity assessment upon cattle arrival, blood sampling at three time points and *Toxoplasma*-specific serological testing using indirect fluorescent antibody tests (IFAT). Results revealed a dynamic pattern of *T. gondii* seropositivity in cattle, with an initial prevalence of 30.6% at arrival (T0) that increased to 44.6% at 14 days (T1) and then decreased slightly to 39.3% at slaughter after 5 months (T2). Interestingly, seroconversion was observed during the study, indicating ongoing infections, and antibody waning occurred in some animals. In terms of blood parameters, seropositive cattle exhibited significantly lower mean corpuscular volume (MCV) and a higher neutrophil–lymphocyte (N/L) ratio, suggesting an activation of the innate immune response. Furthermore, cattle with higher antibody titres displayed higher neutrophil counts. However, all blood parameters with a statistical significance were within the reference range. This study provides for the first time a longitudinal investigation on the serological status for *T. gondii* in naturally exposed beef cattle. These findings provide valuable insights into the clinico-pathological aspects of natural *T. gondii* exposure in cattle and underscore the importance of monitoring and managing *T. gondii* infection in livestock production systems.

**Supplementary Information:**

The online version contains supplementary material available at 10.1007/s00436-024-08189-y.

## Introduction

Toxoplasmosis is a worldwide zoonotic protozoan infection caused by the apicomplexan *Toxoplasma gondii*. Although the disease is usually asymptomatic in the most susceptible species, it can be life-threatening in immunocompromised individuals and can result in abortion or birth of an affected child if a primarily infected woman transmits the parasite to the foetus (Robert-Gangneux and Dardé, 2012; Havelaar et al. 2015). In definitive hosts (Felidae), after a primary infection, sexual replication occurs in small intestine, resulting in the elimination of millions of environmentally resistant oocysts (Dabritz and Conrad 2010). Virtually all warm-blooded species, including humans, can act as intermediate hosts. They will develop bradyzoite tissue cysts, after asexual replication, particularly in muscle and nervous tissues (Dubey 2020). Herbivorous intermediate hosts are of particular importance for the epidemiology of the parasite. By acquiring the infection primarily through the environmental route, they are an indicator of environmental contamination with oocysts and, in the case of livestock species, a source of infection to humans through the consumption of raw/undercooked meat (Tenter et al., 2000, Shapiro et al., 2019). Cattle play a singular role in this parasitosis, with particular host-parasite dynamics and an unclear role in meat-borne transmission. Infection of cattle with *T. gondii* most likely occurs through ingestion of sporulated oocysts spread in pastures and other sources of feed and water (Stelzer et al. 2019). Exposure to the parasite is strongly influenced by livestock husbandry, farm and dietary management. Risk factors linked to infection in this species involve extensive farming systems, the presence of cats and drinking water sources (Gilot-Fromont et al. 2009; Magalhaes et al. 2016). Consequently, seroprevalence rates across Europe range from 7.8 to 83.3% (Klun et al. 2006; Gilot-Fromont et al. 2009; Berger-Schoch et al. 2011; Garcia-Bocanegra et al. 2013; Jokelainen et al. 2017; Blaga et al. 2019, Gazzonis et al. 2020). Interestingly, these seroprevalence rates do not appear to correlate with age, a trend observed in other species (Dámek et al. 2023). There is evidence of the importance of beef consumption in human infection, also in the context of outbreaks (Smith 1993; Baril et al. 1999; Cook et al. 2000; Belluco et al. 2017). Indeed, based on quantitative risk assessment, beef was predicted to be the main source of meat-borne infections in the Netherlands and Italy (Opsteegh et al. 2011a, Bellucco et al. 2018). Unfortunately, in contrast to other species, serological data on *T. gondii* exposure in cattle are of limited use for consumer protection, as no concordance has been shown between the detection of antibodies and the presence of viable tissue cysts (Opsteegh et al. 2011b, 2019). The observed phenomenon can be attributed to the hypothesis that cattle possess the ability to eliminate the parasite, resulting in the development of protective antibody titres, once the parasite has been cleared from their tissues (Opsteegh et al. 2011b). Additionally, despite exposure to the parasite there is little clinical evidence of infection, with only a limited number of congenital transmission cases documented, unlike in small ruminants that often present abortion and symptomatic congenital infection (Canada et al. 2002; Costa et al., 2011; Stelzer et al. 2019). The resistance mechanism observed in cattle infection is thought to be associated with the lethal impact of neutrophil extracellular traps (NETs) on tachyzoites, which merely have an immobilizing effect in sheep (Yildiz et al. 2017). Nevertheless, the specific pathological effects and dynamics of antibody production during natural *T. gondii* infections in cattle remain poorly understood so far.

The aims of this study were to investigate the seroprevalence of *T. gondii* infection at three different stages of the animals’ productive lifespan in a population of beef cattle and to analyse the association between *T. gondii* serological status and blood parameters.

## Materials and methods

### Housing and management

The research was conducted within a commercial fattening facility that housed Limousine bulls imported from France. This facility was situated in the province of Modena, in the Po Valley region of Italy, and the study covered one fattening period that extended from November 2021 to May 2022. The housing system was a semi-closed barn with 44 pens configured in a free stall system. Each pen had the maximum capacity to stock six animals. The pens were arranged in close proximity, separated by iron bars to facilitate interaction among animals in adjacent pens. The floor was slatted, with a pit beneath for manure collection. Prior to introducing the animals, the pens underwent thorough cleaning and disinfection, employing a pressure washer.

A total of 264 animals were delivered to the fattening unit facility in weekly shipments organized in numerically diverse groups, spanning six consecutive weeks (Supplementary Table S3). These animals originated from various farms across France, encompassing different regions within the country. The majority of these bulls were primarily raised either on pastures or in indoor free stall systems with straw bedding.

Before their arrival in Italy, the bulls spent one day in a selection center in France, where they underwent assessments related to their health status, age and body weight. This selection process aimed to create homogeneous groups of animals. Upon their entry into the fattening unit, the bulls were approximately 11 months old and had an average weight of 400 kg. No quarantine period was performed. At the arrival, animals were fed an adaptation diet in order to reduce dietary stressors (Supplementary Table S1).

The production cycle lasted between 5 and 6 months. During this period, 14 bulls were euthanized due to respiratory disease, and 250 bulls finished the cycle and were slaughtered at 600 kg.

### Biosecurity assessment

A biosecurity assessment was performed at arrival to the unit (T0) and 15 days after arrival (T1). An adapted version of the Italian protocol for the assessment of beef cattle welfare included in the ClassyFarm system (Bertocchi et al. 2020) was applied as previously reported (Masebo et al. 2023). The used protocol included a list of 17 items: pests control measures, interaction with other animal species, general precautions to the entrance of occasional visitors, general precautions to the entrance of regular visitors, disinfection of vehicles upon entering the farm, possibility of contact between foreign vehicles and farmed animals (< 20 m), carcass collection (< 20 m), live animal loading, quarantine/housing management, control and prevention of most prevalent infectious diseases, health monitoring activities, control and prevention of endo/ectoparasites, control and analysis of water sources, cleaning of troughs/water point, storage buildings and rooms (hygiene, cleanliness and management of housing environments and bedding) and origin of the drinking water (Supplementary Table S2). For each item, a two- or three-point scale scoring system was applied (1 = insufficient; 2 = acceptable; 3 = optimal). A value for each section was computed by summing the obtained score of each item from each section or area. The obtained values were further converted into percentages. A result below 59% indicated a poor status (= low), a result between 60 and 80% a medium status (= medium) and a result over 80% a good status (= high).

### Haematological investigation

Blood samples from 88 animals were collected by jugular venipuncture for clinical diagnostic investigation at T0 and T1. Two animals were randomly selected from each pen at T0, and the same subjects were again sampled at T1. Samples were transferred to serum vacuum tubes for serological analyses and in EDTA vacuum tubes for complete blood count (CBC) and then to citrate tubes for fibrinogen analysis. The haematological analysis was performed using standard methods on the ADVIA® 120 Haematology System. The following parameters were analysed: RBC, haemoglobin, haematocrit (HCT), mean corpuscular volume (MCV), mean corpuscular haemoglobin (MCH), mean corpuscular haemoglobin concentration (MCHC), red cell distribution width (RDW), platelets (PLT), leukocytes (WBC), neutrophils, monocytes, lymphocytes, eosinophils, basophils and fibrinogen (Supplementary Table S3).

### Sampling at slaughter

Five months after T0, cardiac blood samples were obtained at the slaughterhouse (T2), from 56 of the 88 animals that underwent a blood sampling at T0 and T1. Blood collection took place during the heart excision process, where approximately 10–40 mL of blood was collected in a 50-mL falcon tube and kept at room temperature until further processing.

### Indirect fluorescent antibody test (IFAT)

The blood samples were centrifuged at 750 g for 25 min, and the resulting serum was collected and stored at − 20 °C until further analysis*. T. gondii* indirect fluorescent antibody test (IFAT) for IgG was performed on serum samples. Briefly, slides coated with *T. gondii* tachyzoites (MegaFLUO TOXO-PLASMA g, MegaCor Diagnostik, Hoerbranz, Austria) were probed with 20 μL of serum diluted in phosphate-buffered saline (PBS) with a starting dilution of 1:40. Slides were incubated for 30 min at 37 °C and washed two times with PBS. Internal bovine positive and negative sera controls were included on each slide. The slides were therefore probed with 20 μL of fluorescein isothiocyanate (FITC) conjugated anti-Cattle IgG antibody diluted in PBS at a concentration of 1:200 (Anti-Cattle IgG-FITC antibody, Sigma-Aldrich, Saint Louis, MO) and incubated for 30 min at 37 °C. After two further washing steps with PBS, they were examined under a fluorescent microscope (Dini et al. 2023a). The highest dilution showing fluorescence was the final antibody titre. Serum samples with antibody titre ≥ 1:40 were assessed positive, as 1:40 is the cutoff adopted for diagnostic purpose in different diagnostic facilities in the same area (Dini et al. 2023b).

### Statistical analysis

Data were entered into a statistics program (JMP Pro 17). Descriptive statistics were generated: mean, standard deviation (SD) and/or standard error (SE), median, interquartile range and 95% confidence interval for continuous data, and count and percentage for categorical data. For continuous variables, normality was tested by the Shapiro–Wilk test, and non-normally distributed variables were Box-Cox transformed before the analysis according to previous reports (Raspa et al. 2022). The evaluation of differences between the positive/negative to *T. gondii* and different IFAT titres was undertaken using the Mixed Model Procedure. Each bovine was set as an experimental unit within the arrival group and pen as nested factors (random effect of the model). The seropositive/seronegative status for *T. gondii* (pos/neg) and IFAT titre (1:40/1:80/ > 1:160) within the timepoints were implemented as a fixed effect in separate models. After the analysis, the normal distribution of the data was checked again for the resulting residuals. Means are reported as least square mean, and pairwise multiple comparisons were performed using Tukey test as a post hoc test when a significance was detected. The nominal logistic model was used for categorical variables using the same independent (predictor/explanatory) variables as before mentioned. A *p* value ≤ 0.10 was considered a tendency, a *p* value ≤ 0.05 was considered statistically significant and a *p* value ≤ 0.01 was considered highly significant.

Principal component analysis (PCA) (correlation matrix) was used to reduce the variables to factors as previously reported (Vinassa et al. 2020); data assumption for multivariate normality was checked using Keiser-Meyer-Olkin (KMO) and Barlett tests, which were performed to test the suitability of the data for structure detection.

## Results

### Biosecurity assessment

The biosecurity assessment did not vary between T0 and T1. Biosecurity was scored as medium with a 61% value in both T0 and T1 (Supplementary Table S2).

### Distribution of serological status and IFAT titres for Toxoplasma gondii

**Fig. 1:**
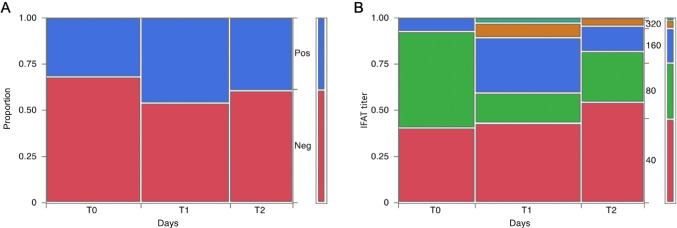
Distribution of serological status for *Toxoplasma gondii* (A) and the IFAT titres in the seropositive cattle (B) at T0, T1 and T2

The distribution of serological status for *T. gondii* is shown in Fig. 1A. The percentage of seropositive animals at T0 was 30.6% and increased at T1 to 44.6%. Finally, at T2, the percentage of seropositive animals was 39.3%. Due to challenges faced during the slaughtering process, the total number of animals collected and tested at T2 was slightly lower (56/88) compared to the numbers obtained during the other two sampling events in the barn.

The distribution of IFAT titres in the seropositive group for *T. gondii* were as follow: at T0 51.2% (1:80) followed by 40.7% (1:40) and 7.4% (1:160) as the maximum titre; at T1, the animals with a titre of 1:40 slightly increased reaching the 43.2% followed by the ones with a titre of 1:160 (29.7%), 1:80 (16.2%), 1:320 (8.1%) and finally 1:1280 (2.7%); finally at T2, the percentage of animals with a titre of 1:40 increased again as the most common category (54.6%), followed by animals with titres > 1:80. Eleven bulls consistently tested positive across all three sampling times, and four of these animals missed a fibrinogen measurement at T0 or T1 (Supplementary Table S3). Additionally, 21 animals consistently tested negative throughout the study. Seroconversion occurred in 13 animals (14.6%) from T0 to T1, and in five (6%) from T1 to T2. No animals lost detectable antibody titre from T0 to T1, while at T2, 12 (14.5%) bulls previously positive (six positive from T0 and six from T1) tested negative for IgG (Fig. 1B).

### Effect of the Toxoplasma gondii serological status on blood analysis

The association between *T. gondii* serological status (positive *vs* negative) and haematological parameters is shown in Table 1. There was a statistically significant association (*p* value < 0.05) with MCV and N/L ratio. MCV was significantly lower, and N/L ratio was significantly higher in seropositive compared with seronegative cattle. However, the MCV mean and N/L ratio median were within the reference range in both seropositive and seronegative animals. There was also a trend effect (*p* value < 0.10) on MCH and neutrophils. MCH and neutrophils tended to be higher in seropositive cattle. The neutrophils median of both the seropositive and the seronegative animals were within the reference range, while the MCH median was below the reference range in both groups.

**Table 1:** Association between the serological status for *Toxoplasma gondii* and the blood analysis

Parameter		*T. gondii* Neg. (*n* = ..)	*T. gondii* Pos. (*n* = …)	*p* value	Reference range
RBC (M/µL)	Mean (SD and SE)	9.82 (1.34; 0.15)	9.71 (1.04; 0.18)	0.65	5.1–7.6^a^
HGB (g/dL)	Mean (SD and SE)	12.07 (1.21; 0.14)	11.94 (1.13; 017)	0.83	8.5–12.2^a^
HCT (%)	Mean (SD and SE)	39.76 (4.31; 0.50)	38.67 (3.66; 0.60)	0.70	22-33^a^
MCV (fL)	Mean (SD and SE)	40.64 (3.06; 0.33)	39.96 (2.37; 0.37)	0.04	38-41^a^
MCH (pg)	Median [CI: 5; 25; 75; 95]	12.20 [10.9; 11.6; 13.1; 14.7]	12.40 [10.8; 11.7; 13.0; 13.9]	0.07	14-18^a^
MCHC (g/dL)	Mean (SD and SE)	30.45 (1.33; 0.15)	30.76 (1.10; 0.18)	0.13	34-38^b^
RDW (%)	Mean (SD and SE)	24.06 (1.84; 0.22)	24.06 (1.86; 0.24)	0.91	15.5–19.4^a^
PLT (K/µL)	Median [CI: 5; 25; 75; 95]	283 [48; 150; 409; 789]	151.5 [22; 152; 428; 891]	0.29	193-637^a^
WBC (K/µL)	Median [CI: 5; 25; 75; 95]	9.24 [6.3; 7.8; 11.7; 16.4]	9.29 [5.4; 7.7; 10.6; 17.0]	0.72	4.9-12a
NEU (K/µL)	Median [CI: 5; 25; 75; 95]	3.42 [1.8; 2.8; 4.7; 9.0]	3.77 [2.2; 2.6; 8.6; 9.8]	0.08	1.8–6.3^a^
MONO (K/µL)	Median [CI: 5; 25; 75; 95]	1.23 [0.5; 1.0; 1.5; 2.1]	1.10 [0.5; 0.9; 1.4; 1.7]	0.38	0–0.6^a^
LYM (K/µL)	Mean (SD and SE)	4.30 (1.64; 0.18)	3.93 (1.42; 0.22)	0.20	1.6–5.6^a^
EOS (K/µL)	Median [CI: 5; 25; 75; 95]	0.13 [0.03; 0.05; 0.29; 1.45]	0.10 [0.03; 0.06; 0.30; 0.68]	0.59	0–0.9^a^
BASO (K/µL)	Median [CI: 5; 25; 75; 95]	0.07 [0.03; 0.05; 0.10; 0.22]	0.07 [0.02; 0.05; 0.11; 0.30]	0.93	0–0.3^a^
FIBR (mg/dL)	Median [CI: 5; 25; 75; 95]	916.2 [433; 662; 1348; 1507]	826.5 [389; 636; 1112; 1494]	0.28	100-600^b^
N/L ratio	Median [CI: 5; 25; 75; 95]	0.85 [0.33; 0.59; 1.18; 6.13]	1.04 [0.47; 0.69; 1.74; 3.03]	0.03	0.4–2.34^a^

Abbreviations: *Neg* negative, *Pos* positive, *RBC* red blood cell, *HGB* haemoglobin, *HTC* haematocrit, *MCV* mean corpuscular volume, *MCH* mean corpuscular haemoglobin, *MCHC* mean corpuscular haemoglobin concentration, *RDW* red blood cell distribution width, *PLT* platelets, *NEU* neutrophils, *WBC* white blood cells, *MONO* monocytes, *LYM* lymphocytes, *EOS* eosinophils, *BASO* basophils, *FIBR* fibrinogen, *N/L ratio* neutrophils:lymphocytes ratio, *M/µL* 10^6^ per microliter, *%* percentage, *K/µL* 10^3^ per microliter, *g/dL* grams per deciliter, *fL* femtoliter, *pg* picogram, *mg/dL* milligram per decilitre

^a^George et al. (2010)

^b^Cornell University College of Veterinary Medicine (2023)

### Effect of different IFAT titres of Toxoplasma gondii on blood analysis

There was a significant effect association (*p* value < 0.05) of the different titres with neutrophils and N/L ratio (Table 2). The neutrophils and consequently the N/L ratio were significantly higher in cattle with titres > 1:160 compared to cattle with titres of 1:80 and 1:160. However, the neutrophils and N/L ratio medians were within the reference range in animals presented cattle with titres of 1:80, 1:160 and > 1:160.

**Table 2:** Association between different serological titre *Toxoplasma gondii* and the blood analysis

Parameter		40	80	> 160	*p* value	Reference range
NEU (K/µL)	Median [CI: 5; 25; 75; 95]	3.37 [2.0; 2.6; 3.9; 7.1]	4.01 [2.1; 2.9; 4.6; 6.99]	5.39 [2.4; 3.8; 8.2; 17.0]	0.03	1.8–6.3^a^
N/L ratio	Median [CI: 5; 25; 75; 95]	0.65 [0.4; 0.9; 1.2; 2.7]	0.85 [0.4; 0.6; 1.2; 3.1]	1.02 [0.4; 2.0; 1.4; 12.9]	0.02	0.4–2.34^a^

Abbreviations: *NEU* neutrophils, *N/L ratio* neutrophils:lymphocytes ratio, *K/µL* 10^3^ per microliter

^a^George et al. (2010)

### Principal component analysis

**Fig. 2:**
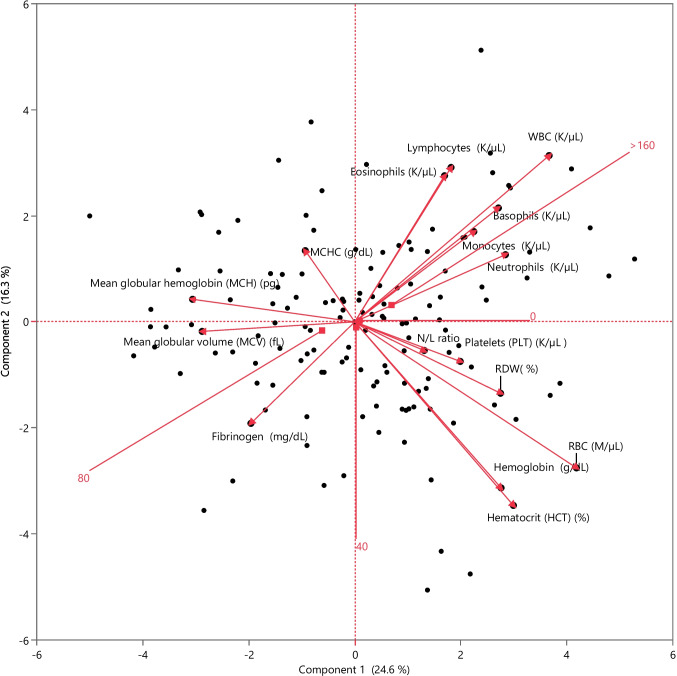
Principal component analysis biplot (PC1 and PC2) performed on blood parameters according to serological status for *Toxoplasma gondii*

PCA was performed to explain the variability in the blood analysis and to correlate each blood parameter according to serological status for *T. gondii*. The suitability of the data for PCA was evaluated (KMO = 0.80; Barlett’s test, *p* < 0.01). Figure 2 shows that PCA separated the blood parameters on the first principal component (PC1): component 1 explains 24.6% of the variance of the data, and component 2 (PC2) another 16.3%, for a total of 40.9% of variability of variance. Table 3 shows the loadings of the variables of the first and second principal components, and how each variable contributes to each component. Even though the obtained PCA showed a small effect, three different patterns were identified. Cattle with titres > 1:160 showed a greater number of eosinophils, lymphocytes, WBC, basophils, monocytes and neutrophils, while cattle with a titre of 1:80 showed a correlation with MCV and fibrinogen. Finally, cattle with titres of 1:40 or seronegative showed no effect on the considered blood parameters.

**Table 3:** Principal component analysis loadings of blood parameters according to serological status for *Toxoplasma gondii*

Parameter	PCA1 (24.6%)	PCA2 (16.3%)
RBC (M/µL)	39.9%	− 32.3
HGB (g/dL)	26.3%	− 36.7%
HCT (%)	28.6%	− 40.6%
MCV (fL)	− 27.5%	− 2.1%
MCH (pg)	− 29.2%	5%
MCHC (g/dL)	− 8.9%	15.7%
RDW (%)	26.3%	− 15.8%
PLT (K/µL)	19%	− 8.7%
WBC (K/µL)	35%	36.8%
NEU (K/µL)	27.1%	14.9%
MONO (K/µL)	21.4%	20%
LYM (K/µL)	17.3%	34.1%
EOS (K/µL)	16.2%	32.3%
BASO (K/µL)	25.9%	25.2%
FIBR (mg/dL)	− 18.7%	− 22.4%
N/L ratio	12.5%	− 6.3%

Abbreviations: *RBC* red blood cell, *HGB* haemoglobin, *HTC* haematocrit, *MCV* mean corpuscular volume, *MCH* mean corpuscular haemoglobin, *MCHC* mean corpuscular haemoglobin concentration, *RDW* red blood cell distribution width, *PLT* platelets, *NEU* neutrophils, *WBC* white blood cells, *MONO* monocytes, *LYM* lymphocytes, *EOS* eosinophils, *BASO* basophils, *FIBR* fibrinogen, *N/L ratio* neutrophils:lymphocytes ratio, *M/µL* 10^6^ per microliter, *%* percentage, *K/µL* 10^3^ per microliter, *g/dL* grams per deciliter, *fL* femtoliter, *pg* picogram, *mg/dL* milligram per decilitre

## Discussion

The present study consisted of a longitudinal investigation on the serological status for *T. gondii* in beef cattle naturally exposed at three different stages of the productive cycle. We observed a relatively high seroprevalence of *T. gondii* infection among cattle at T0, with 30.6% of the animals tested positive. This initial prevalence suggest that animals were already infected at arrival to the fattening unit. However, the most noteworthy finding was the increase in seropositivity observed at T1, where 44.6% of the cattle tested positive for *T. gondii* antibodies. Considering the kinetics of IgG, this rise in seropositivity at T1 implies that some cattle may have become infected at the time of arrival in the fattening unit, during the transport or immediately before the shipping. Furthermore, we identified a subset of animals (five in total) that underwent seroconversion from T1 to T2, indicating that these individuals likely acquired the infection during their time in the fattening unit rather than before their arrival. Interestingly, when comparing T1 with T2, a slight decrease in seroprevalence at T2 was noticed. This reduction suggests that some cattle may have lost detectable antibody titres by the time of slaughter, potentially indicating a waning of the immune response or clearance of the infection in these individuals. Overall, these findings highlight the dynamic nature of *T. gondii* infection in fattening cattle, with evidence of both new infections and antibody waning over the course of their productive cycle.

Estimates of seroprevalence in cattle, when obtained by highly specific assays, may be useful for monitoring exposure of bovines to *T. gondii*. Different serological techniques have been recommended and considered suitable for the confirmation of exposure to *T. gondii* in cattle such as IFAT, modified agglutination test (MAT) and enzyme linked immunosorbent assay (ELISA) (World Organisation For Animal Health – OIE 2017). Nevertheless, results of seropositivity should be interpreted with caution, as studies using bioassay experiments on naturally exposed cattle indicate that the overwhelming majority of seropositive cattle do not show evidence of viable *T. gondii* infection (Boch et al. 1965; Dubey et al. 2005; Dubey and Streitel 1976; Jacobs and Moyle 1963; Opsteegh et al. 2019). On the contrary, there are a limited number of studies of naturally exposed cattle in which positive *T. gondii* bioassays indicate viable infection (Arias et al. 1994; Catar et al. 1969; de Macedo et al. 2012; Dubey 1992; Jacobs et al. 1960). Therefore, identification of *T. gondii* genomic material without positive bioassays should not be considered conclusive of infection and consequently does not provide an indication of risk for the consumer (Opsteegh et al. 2019; Stelzer et al. 2019).

Herein within the seropositive group, the most prevalent IFAT titre at T0 was 1:80, and at T1 and T2 was 1:40. Moreover, seroconversion occurred in 14.6% of cattle from T0 to T1, and in 6% from T1 to T2. No animals lost detectable antibody titre from T0 to T1, while at T2 14.5% became seronegative for IgG. Among these, half were already IgG positive at T0, while the remaining half became positive at T1. Therefore, our results show that antibody levels to *T*. *gondii* in cattle are variable. This may suggest infection and a capability to eliminate the infection, leading to a decrease in antibody levels. However, it is important to consider the possibility of serological cross-reactions among various Sarcocystidae, which could influence these results.. The host-*T. gondii* interaction in cattle is poorly understood, and only few studies have investigated the antibody kinetics (Dubey et al., 1985; Opsteegh et al. 2011a). It has been observed that the dynamics of anti-*T. gondii* antibody levels in cattle are influenced by age, with infected adult cattle typically exhibiting low antibody titres (Dubey et al., 1985). Additionally, it could be postulated that calves exposed to low doses of parasite early in life, which may result in the generation of a relatively weak protective immunological response, could experience reversion to seronegative status during their relatively short lifespan, as observed in our case (16–18 months).

The fact that an increase in seroprevalence was observed during the 5 months of the fattening period may indicate that some risk factors may have been present in the investigated fattening farm leading to *T. gondii* infection of the cattle. In the biosecurity assessment, the main risk factors identified were inadequate rodent and insect control measures, the possibility of contact with other animal species such as cats and the possibility of contamination of drinking water in case of failure of the central water supply (use of a storage tank). The presence of cats (Gilot-Fromont et al. 2009; Magalhaes et al. 2016; Sun et al. 2015) and rodents (Sun et al. 2015) on farms are considered important risk factors for infection of cattle. In addition, access to water from a reservoir has also been identified as a potential risk factor (Magalhaes et al. 2016).

In respect to the blood parameters, MCV was significantly lower, and N/L ratio was significantly higher in seropositive compared with seronegative cattle and the MCH and neutrophils tended to be higher in seropositive cattle. Furthermore, the neutrophils and consequently the N/L ratio were also significantly higher in cattle with titres > 1:160 compared to cattle with lower titres. However, all blood parameters were within the reference range. Based on our results, it is not possible to confirm that neutrophils are associated with the activation of the innate immune response in *T. gondii* positive animals, although a paraphysiological increase in neutrophils is observed in animals with higher titres. An effective innate immune response plays a crucial role in the early recognition of *T. gondii* (Wilson 2012). Neutrophils, essential components of the innate immune system, are produced in the bone marrow, and despite their relatively short lifespan, they rapidly accumulate at the site of infection employing diverse strategies to fight invading pathogens (Mantovani et al. 2011). Their primary function revolves around phagocytosis, wherein pathogens taken up by neutrophils are subsequently eradicated within the phagolysosome through the actions of enzymes and proteins (Brinkmann and Zychlinsky 2012). Neutrophilia, a common occurrence during numerous infections, was observed in our study in association with seropositivity and higher antibody titres. This phenomenon may be linked to recent toxoplasmic infections, where IgGs have yet to be fully developed. A limitation of our study lies in the absence of IgM research, which is more closely related to the acute phase of infection. In the context of toxoplasmosis, neutrophils appear to hold particular significance. *T. gondii* tachyzoites have been shown to induce the formation of extracellular trap structures in murine and human neutrophils (NETs) (Abi Abdallah et al. 2011; Manda et al. 2014). These extracellular traps not only physically entrap tachyzoites, preventing host cell invasion, but also exert detrimental effects on the viability of the trapped tachyzoites (Abi Abdallah et al. 2011). Furthermore, it was demonstrated that NET structures released from sheep polymorphonuclear leukocytes (PMNs) led to the mechanical immobilization of *T. gondii* tachyzoites, while NET structures released from cattle PMNs appeared to have lethal effects on the tachyzoites (Yildiz et al. 2017). It might be premature and speculative to establish a direct link between our research findings and the role of NETosis in bovine toxoplasmosis. However, our study does provide valuable insights suggesting a discernible association between neutrophils and seropositivity for *T. gondii* antibodies in cattle. This observation contributes to a better understanding of the clinico-pathological aspects of naturally exposure of *T. gondii* in cattle.

## Conclusion

This study evaluates for the first time the antibody kinetics for *T. gondii* at three different time points of the production cycle of fattening cattle. The high seroprevalence of *T. gondii* infection among cattle at T0, with an increase in T1 and a slight decrease in T2 emphasize the dynamic nature of *T. gondii* infection in cattle, with evidence of both new infections and antibody decay during the production cycle. In addition, it was observed that the same titre varies in different samplings over time, suggesting that this species has a peculiar antibody dynamic.

Regarding blood parameters, animals with higher titres showed a slight increase in neutrophil levels, which could be considered a paraphysiological innate immune response in the *T. gondii* seropositive animals. However, further studies are needed to better understand the specific behaviour of neutrophils in cattle exposed to *T. gondii*.

### Supplementary Information

Below is the link to the electronic supplementary material.

Supplementary file1 (DOCX 16 KB)

Supplementary file2 (DOCX 26 KB)

Supplementary file3 (XLSX 5708 KB)

## Data Availability

Not applicable.
