# Supplementary material for: Observational longitudinal study on Toxoplasma gondii infection in fattening beef cattle: serology and associated haematological findings
Source: Parasitol Res. 2024 Mar 23;123(3):169. doi: 10.1007/s00436-024-08189-y (PMC10960757; doi:10.1007/s00436-024-08189-y)
Supplement: Supplementary file 1 — Supplementary file1 (DOCX 16 KB) [file 436_2024_8189_MOESM1_ESM.docx]

**Observational longitudinal study on *Toxoplasma gondii* infection in fattening beef cattle: serology and associated haematological findings**

Filippo M. Dini *^a1^, Joana G.P. Jacinto ^a1^, Damiano Cavallini ^a^, Andrea Beltrame ^b^, Flavia S. Del Re ^a^, Laura Abram ^a^, Arcangelo Gentile ^a^, Roberta Galuppi ^a^

Supplementary Table S1. Analysis of the adaptation TMR diet (T0 and T1) and chemical analysis.

| **TMR** | **Feed, kg af** |
| --- | --- |
| Wheat silage | 3.5 |
| Meadow hay^1^ | 1.2 |
| Wheat straw | 1.1 |
| Beat pulp | 1.3 |
| Corn, finely ground^2^ | 1.1 |
| Soybean meal | 0.5 |
| Cane molasses^3^ | 0.5 |
| Min&Vit Premix | 0.3 |
|  | **Nutrients, %DM** |
| DM | 70.07 |
| UFC | 0.81 |
| CP^4^ | 11.25 |
| Ash | 8.78 |
| EE^5^ | 2.06 |
| Starch | 13.57 |
| Sugars | 7.37 |
| NDF^6^ | 39.21 |
| ADF^7^ | 25.79 |
| ADL^8^ | 3.45 |

^1^ the quality of the hay was checked to ensure the absence of molds and spores (Cavallini et al. 2022). ^2^ the corn was below the EU maxim tolerable level (Girolami et al. 2022). ^3^ molasses were properly characterized (Palmonari et la. 2021). ^4^ Crude protein. ^5^ ether extract. ^6^ neutral detergent fiber. ^7^ acid detergent fiber. ^8^ acid detergent lignin.

Cavallini D, Penazzi L, Valle E, Raspa F, Bergero D, Formigoni A, Fusaro I. 2022. When changing the hay makes a difference: A series of case reports. J Equine Vet Sci. 113:103940.

Girolami F, Barbarossa A, Badino P, Ghadiri S, Cavallini D, Zaghini A, Nebbia C. 2022. Effects of turmeric powder on aflatoxin M1 and aflatoxicol excretion in milk from dairy cows exposed to aflatoxin b1 at the EU maximum tolerable levels. Toxins 14: 430.

Palmonari A, Cavallini D, Sniffen CJ, Fernandes L, Holder P, Fusaro I, Giammarco M, Formigoni A, Mammi LME. 2021. In vitro evaluation of sugar digestibility in molasses. Ital. J. Anim. Sci. 20:571-577.
