# Supplementary material for: Observational longitudinal study on Toxoplasma gondii infection in fattening beef cattle: serology and associated haematological findings
Source: Parasitol Res. 2024 Mar 23;123(3):169. doi: 10.1007/s00436-024-08189-y (PMC10960757; doi:10.1007/s00436-024-08189-y)
Supplement: Supplementary file 2 — Supplementary file2 (DOCX 26 KB) [file 436_2024_8189_MOESM2_ESM.docx]

**Observational longitudinal study on *Toxoplasma gondii* infection in fattening beef cattle: serology and associated haematological findings**

Filippo M. Dini , Joana G.P. Jacinto , Damiano Cavallini , Andrea Beltrame , Flavia S. Del Re , Laura Abram , Arcangelo Gentile , Roberta Galuppi

**Supplementary Table S2.** Check-list used to perform the biosecurity assessment.

|  |  | | **T0** | **T1** |
| --- | --- | --- | --- | --- |
|  | **AREA BIOSECURITY** | |  |  |
|  | **Items** | **Level** |  |  |
| 1. | Rodent and insect control measures | Total absence of control measures |  |  |
|  |  | Presence of rough and minor control measures (absence of written plans) | 2 | 2 |
|  |  | Presence of defined and effective procedures supported by written manual and recording system |  |  |
| 2. | Contact with other animal species | Yes, the contact is frequent and evident |  |  |
|  |  | No, contact may occur but is not evident at the time of the visit such as cats | 2 | 2 |
|  |  | No, the farm is well protected (fences, etc.); no other animal species are present on the farm perimeter, and no contact with herds of the same species or other animals |  |  |
| 3 | General precautions at the entrance of occasional visitors | Total absence of measures |  |  |
|  |  | Presence of minor procedures (absence of written plans) | 2 | 2 |
|  |  | Presence of defined and effective procedures supported by written and recording system manual |  |  |
| 4 | General precautions at the entrance of regular visitors | Total absence of measures |  |  |
|  |  | All visitors are required to wear disposable footwear before entering the farm or use boots that are on the farm for their exclusive personal use | 2 | 2 |
|  |  | All visitors must pass through a changing area and are required to wear disposable footwear and clothes provided by the farm or use clothing and boots that remain on the farm for their exclusive personal use |  |  |
| 5. | Disinfection of vehicles upon entering the farm | Absence of disinfection facilities | 1 | 1 |
|  |  | Presence of non-specific disinfection facilities or used of disinfection aids only when necessary |  |  |
|  |  | Presence of specific, fixed and routinely used disinfection facilities |  |  |
| 6. | Possibility of contact between foreign vehicles and farmed animals (< 20 m) | Yes | 1 | 1 |
|  |  | No |  |  |
| 7. | Carcass collection (< 20 m) | Yes, vehicles used to remove the carcasses have direct/indirect contact with cattle (< 20 m distance) | 1 | 1 |
|  |  | No, vehicles used to remove the carcasses are stopped at the border of the farm w (>20m distance) |  |  |
| 8. | Live animal loading (i.e. for sale) | Loading is carried out close to the housing premises where the animals are kept (<20 m) | 1 | 1 |
|  |  | Loading is carried out away from the housing premises where the animals are kept (>20 m) |  |  |
| 9. | Quarantine/Housing management | No quarantine for new entering animals | 1 | 1 |
|  |  | Partial/minor quarantine measures (i.e. designated area not separated from the areas where the other cattle are kept, quarantine is too short, no biological tests) |  |  |
|  |  | Proper quarantine measures, adequate in time and facilities (i.e. designated area separated from the areas where the other cattle are kept, adequate duration of the quarantine, biological tests) |  |  |
| 10. | Control and prevention of most prevalent infectious diseases | No knowledge of most prevalent infectious diseases or no information of the herd health status |  |  |
|  |  | Partial knowledge and/or presence of undefined plans (i.e., approximate, random, and not continuous over time) |  |  |
|  |  | Knowledge of at least three diseases prevalence in the herd; in addition, application of proper operational plans of prevention and control on at least two of them (vaccination plan, plan for dealing of infected animals, eradication plan, etc.). | 3 | 3 |
| 11. | Health monitoring activities (Verify the farm's habit of submitting pathological material, fetuses, carcasses, and blood samples to the reference testing laboratory; the farmer must be in possession of an analytical result from the last 12 months) | Absence |  |  |
|  |  | Presence of analysis on pathological material | 2 | 2 |
| 12. | Control and prevention of endo/ectoparasites | No knowledge and absence of prevention/control plans |  |  |
|  |  | Partial knowledge and/or presence of random control and prevention plans (i.e. approximate, random, and not continuous over time) |  |  |
|  |  | Knowledge of most prevalent parasites on the farm and prevention performed following laboratory tests | 3 | 3 |
| 13. | Control and analysis of water sources | Absence of water analysis |  |  |
|  |  | Drinking water comes from the central supply system or from other sources and the quality of the water is checked at least once a year | 2 | 2 |
| 14. | Cleaning of troughs/water point | Presence of dirt on the surface and walls of troughs/water point |  |  |
|  |  | Presence of food only on the water surface or only on the bottom. The water still remains clear | 2 | 2 |
|  |  | Absence of dirt, clean troughs/water point and clear water |  |  |
| 15. | Storage buildings and rooms: hygiene, cleanliness and management of housing environments and bedding | Inadequate: Dirty, unmanaged and/or animal-harmful housing and bedding environments |  |  |
|  |  | Adequate: Fairly clean and sufficiently managed housing and/or bedding environments and/or clean grid in almost all groups | 2 | 2 |
|  |  | Optimum: Clean, dry and optimally managed housing and bedding environments with frequent material changes |  |  |
| 16. | Origin of the drinking water | only one drinking water source and no storage tank |  |  |
|  |  | Only one drinking water source but presence of a storage tank that guarantees a sufficient water supply in case of disruption of the water source | 2 | 2 |
|  |  | presence of two or more drinking water sources |  |  |
| TOTAL obtained | | | 27 | 27 |
| TOLTAL obtained in percentage | | | 61% | 61% |

**Abbreviations:** T0 = Assessment at day 2 after arrival; T1=Assessment at day 15 after arrival.
